# Results of a study on the correlation between medical students’ adaptability and stress

**DOI:** 10.1192/j.eurpsy.2026.12089

**Published:** 2026-07-31

**Authors:** P. Sarantuya, B. Purev, K.-U. Mijgee, B. Dolgorsuren, T. Myatav

**Affiliations:** 1 Graduate School of Education, Graduate University of Mongolia; 2 Medical Department, School of Medicine, Etugen University; 3 Department оf Psychology аnd Methodology, School оf Educational Studies, Mongolian National University of Education, Ulaanbaatar, Mongolia

## Abstract

**Introduction:**

Stress is one of the important factors that affects students’ academic performance. Medical students are constantly exposed to pressure, such as academic load and exams, which makes students with weak adaptive capacity more susceptible to stress, and, to cope with stress, they make various changes in their daily lives, such as insomnia, irregular eating, smoking, and drug use. Inability to cope with stress effectively leads to poor academic and professional performance and increases psychological distress. Therefore, research has shown that exposure to stress is higher during medical school. First-year students are more likely to be exposed to stress due to the lack of adequate adaptation to the learning environment, learning process, social interaction, socio-psychological protection, support, and success needs.

**Objectives:**

To investigate the relationship between the adaptability of first-year students at the School of Health Sciences of Etugen University and some of the factors that influenced it.

**Methods:**

The Mental Health Screening Questionnaire—SRQ-20 is a 20-item self-report questionnaire developed by WHO specifically for primary health care services. In order to study how students’ learning motivation is related to the adaptive process, the T.I. Ilyin University Motivation Assessment Methodology was used to detect learning motivation in three areas: acquiring knowledge, acquiring a profession, and obtaining a diploma.

**Results:**

402 first-year students aged 16 to 31 from the School of Medicine of Etugen University participated. Of the participants, 74.39% were stressed, 43.29% were stressed, and 31.1% were classified as having a stressful and possibly problematic psychobehavioral condition, which requires consideration, review, and research. When the stress score increases by one, adaptation to learning activities increases by 27.46% (p=0.023), and when the F48 fatigue score increases by one, adaptation to learning activities decreases by 35.7% (p=0.045), which is a negative effect, and r=-0.48 indicates that these two indicators are inversely correlated. According to the adaptation indicators among the school community, 73.63% are at an average level of adaptation, and the indicators in the professional field are similar. The high adaptation indicator of 33.08% to learning activities is 18.65% higher than the adaptation ability score among the school community. When the adaptation score among the learning environment and community increases by one, the adaptation score to learning activities increases by 58.2%, and P=0.000 is more significant. Stress scores are correlated with adaptability scores (r=0.58, p=0.00).

**Conclusions:**

Adaptation to the learning environment and adaptation to learning activities are at an average level among first-year students. It also indicates that students are exposed to stress in their first year of university and need psychological help and support.

**Disclosure of Interest:**

None Declared

